# Safety and effects of volume loading during transesophageal echocardiography in the pre-procedural work-up for left atrial appendage closure

**DOI:** 10.1186/s12947-020-00230-1

**Published:** 2021-01-02

**Authors:** Afonso B. Freitas-Ferraz, Mathieu Bernier, Kim O’Connor, Jonathan Beaudoin, Jean Champagne, Jean-Michel Paradis, Gilles O’Hara, Guillem Muntané- Carol, Alberto Alperi, Laurent Faroux, Lucia Junquera, Josep Rodés-Cabau

**Affiliations:** Department of Cardiology, Quebec Heart & Lung Institute, Laval University, 2725, chemin Sainte-Foy, Quebec City, G1V 4G5 Quebec Canada

**Keywords:** Left atrial appendage, Left atrial appendage closure, Watchman, Amplatzer, Ultraseal, Transesophageal echocardiography, Volume loading

## Abstract

**Background:**

In patients undergoing left atrial appendage (LAA) closure, an accurate sizing of the LAA is key to optimize device sizing, procedural success and reduce complications. Previous studies have shown that intraprocedural volume loading increases LAA dimensions and improves device sizing. However, the safety and effects on LAA and device sizing of administering a fluid bolus during pre-procedural transesophageal echocardiography (TEE) are unknown. The aim of this study was to determine the safety and impact on LAA dimensions and device sizing of an intravenous (IV) fluid bolus administered during TEE in the setting of the pre-procedural work-up for LAA closure.

**Methods:**

The study included a total of 72 patients who underwent TEE to assess suitability for LAAC and received a 500 ml IV bolus of normal saline. The LAA landing zone (LZ) and depth were measured by TEE before and after volume loading, and these measurements were used to predict the device size implanted during a subsequent percutaneous LAAC procedure.

**Results:**

There were no complications associated with volume loading. The baseline mean LZ was 19.6 ± 3.6 mm at 90^o^, and 20.2 ± 4.1 mm at 135^o^. Following fluid bolus, the maximum diameter increased 1.5 ± 1.0 mm at 90^o^ (*p*<0.001), and 1.3 ± 1.0 mm at 135^o^ (*p*<0.001). The baseline mean depth of the LAA was 26.5 ± 5.5 mm at 90^o^, and 23.9 ± 5.8 mm at 135^o^. After fluid bolus, the mean depth increased by 1.5 ± 1.8 mm (*p*<0.001) and 1.6 ± 2.0 (*p*<0.001), at 90^o^ and 135^o^, respectively. Sizing based on post-bolus measurements of the LZ significantly improved the agreement with the final device size selection during the procedure in 71.0% of cases (vs. 42.0% with pre-bolus measurements).

**Conclusions:**

Volume loading during ambulatory TEE as part of the pre-procedural work-up of LAAC is safe and significantly increases LAA dimensions. This strategy may become the new standard, particularly in centers performing LAAC with no TEE guidance, as it improves LAA sizing and more accurately predicts the final device size.

## Background

Atrial fibrillation (AF) is the most common sustained cardiac rhythm disorder encountered in clinical practice and poses a major public health concern with high morbidity [1]. The most debilitating complication of AF is stroke, which is more severe, more likely to recur and is associated with a higher mortality than non-AF related strokes [2, 3]. Oral anticoagulant effectively reduces the risk of ischemic stroke and systemic thromboembolism, and is considered the preferred therapy for most patients with AF [4, 5]. Nevertheless, in those deemed poor candidates for long-term anticoagulation, percutaneous left atrial appendage closure (LAAC) provides a valid therapeutic alternative [4–6].

Currently, LAA sizing relies mainly on transesophageal echocardiography (TEE) measurements that are performed under fasting conditions, which may lead to a decrease in intravascular volume. Since the LAA is a compliant structure that responds to volume loading, sizing the LAA in these conditions may underestimate its true dimensions. Indeed, previous data have shown that administering 500–1000 mL of normal saline during the procedure is associated with an average increase of 10% in the width and depth of the LAA [7, 8]. However, in the aforementioned studies, volume loading was performed intraprocedurally, in patients under general anesthesia and invasive hemodynamic monitoring. Moreover, some centers perform LAAC under fluoroscopic guidance only, and there has been a growing trend towards the use of intracardiac echocardiography as a potential alternative to TEE that mitigates the need for general anesthesia and expedite procedural logistics [9–11]. In these scenarios, an accurate preprocedural LAA sizing using TEE becomes even more important to optimize results and reduce complications related with inaccurate device sizing.

The aim of the present study was to determine the safety and impact on LAA dimensions and device sizing of an intravenous (IV) fluid bolus administered in the ambulatory setting, during the pre-procedural work-up for patient suitability for LAAC.

## Methods

From January 2017 to February 2020, a total of 72 patients with AF who underwent an ambulatory TEE as part of the pre-procedural work-up for LAAC received an IV bolus of normal saline to ensure adequate volume loading. Indications for LAAC and the choice of the occluder device to be implanted in a subsequent scheduled intervention were based on the assessment of a multidisciplinary team, including interventional cardiologists, electrophysiologists and echocardiographists. All patients provided informed consent for the procedures and Ethics committee approval was obtained for data collection and analysis.

### Echocardiographic study

All baseline TEE examinations were performed under conscious sedation, with midazolam alone or in combination with fentanyl, by an experienced cardiologist. Heart and respiratory rate, oxygen saturation and non-invasive blood pressure were monitored at baseline, and every 5 minutes throughout the duration of the procedure.

A multiplane TEE probe (Philips iE33 (X7-2t) or GE Vivid E9 (6VT-D)) was used to assess LAA morphology, landing zone (LZ) dimensions and the maximum depth of the dominant lobe, before and after the administration of 500 mL IV bolus of normal saline. For purposes of consistency and simplification, the LZ and depth were measured at 90° and 135°, since these angles usually yield the largest dimensions [7]. The saline infusion was administered during or immediately after TEE probe insertion, and did not significantly increase procedural time since it takes place while standard steps are being performed. As per hospital protocol, during the time it takes for the bolus to be administered, we performed an evaluation of the heart valves and left ventricular function, leaving the detailed assessment of the LAA for last.

**Fig. 1:**
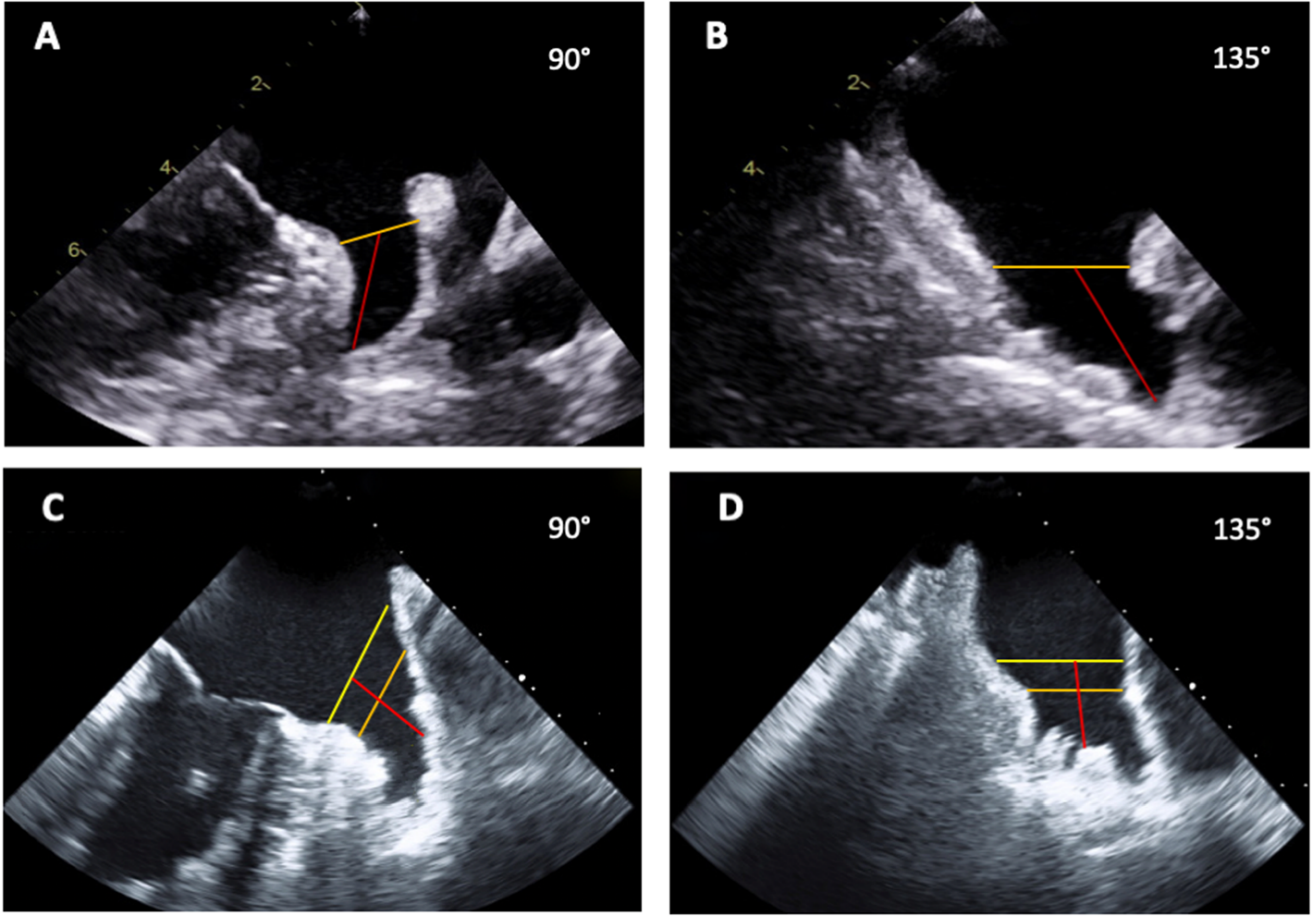
Transesophageal echocardiography measurements for left atrial appendage closure using the Watchman device and the the Amplatzer Cardiac Plug/Ultraseal devices. For the Watchman device (**a/b**), the LZ (orange line) measurement is taken from the circumflex artery medially to 1-2 cm laterally inside the limbus, and the depth (red line) is measured from the LZ to the most distal tip of the main lobe. For the Amplatzer or the Ultraseal devices (**c/d**), the orifice (yellow line) is the line that connects the circumflex artery to the pulmonary vein ridge, and the LZ (orange line) is measured at 10-12 mm within the LAA orifice .The depth (red line) is measured from the orifice to the back wall of the LAA in a perpendicular line. LAA: left atrial appendage; LZ: landing zone

Images were digitally stored and retrospectively analyzed according to the device type that was finally implanted:

- For the Watchman device (Boston Scientific, Natick, Massachusetts), the LZ was measured from the circumflex artery to a superior point 15 mm within the pulmonary vein ridge (PVR); and the LAA depth was measured from the landing zone to the most distal tip of the main lobe (Fig. 1a and b);

- For the Amplatzer Cardiac Plug (AGA Medical Corporation, Golden Valley, MN) and the Ultraseal devices (Cardia, Eagan, Minnesota), the LAA orifice was measured from the PVR to the junction of the left atrium with the LAA at the level of the circumflex artery. The LZ was then measured at 10 mm within the LAA orifice and parallel to the line that defined the LAA orifice. Lastly, the depth was measured from a line that runs perpendicularly from the orifice to the posterior wall of the LAA (Fig. 1c and d).

All percutaneous LAA closure procedures were performed in a subsequent intervention (1–3 weeks later) under general anesthesia and TEE guidance. During LAA closure, intraprocedural volume loading was adapted to each patient volume status. The LA pressure was checked after transseptal puncture, and fluids were given to ensure a LA pressure >10–12 mmHg. LAA measurements were performed before contrast administration.

The predicted device size based on the widest diameter of the LZ (as per the manufacturer’ recommendations), before and after volume loading, was compared to the device that was actually implanted.

### Statistical analysis

Categorical variables were expressed as a number (percentage) and continuous variables as mean (standard deviation) or median (interquartile range [IQR]: 25-75th percentile), according to their distribution. Assessment of normality for continuous data was performed using the Shapiro-Wilks test. Quantitative variables were analyzed with a paired Student t-test. All tests were 2-sided, and *p* values < 0.05 were considered significant. A simple regression model was used to investigate the influences of age, sex and body surface area on post volume loading LAA dimensions (delta). The analyses were performed using STATA (version 14.2; StataCorp LLC, College Station, Tx, USA). The data underlying this article will be shared on reasonable request to the corresponding author.

## Results

Baseline clinical demographics of the study population are summarized in Table 1. The mean age of the patients was 75 ± 8 years, 29% were women and 15% had reduced left ventricular ejection fraction (LVEF<40%). The average CHA_2_DS_2_-VASc and HASBLED score was 4.3 ± 1.6 and 3.7 ± 0.9, respectively. In two patients (2.8%) a pediatric TEE probe was used: one due to resistance encountered during probe insertion/advancement, and the other due to prior esophageal disease. All TEE procedures were performed without any complications and the administration of 500 mL IV bolus of normal saline was well tolerated by all patients.

**Table 1:** Baseline clinical demographics of the study population

	Study Population (***n***=72)
Age (years)	75.2±7.7
Female	21 (29.2)
BMI (kg/m^2^)	29.4±8.7
Diabetes mellitus	27 (37.5)
Hypertension	62 (86.1)
Peripheral artery disease	9 (12.5)
Coronary artery disease	32 (44.4)
Prior myocardial infarction	8 (11.1)
Prior CABG	13 (18.1)
Heart failure history	13 (18.1)
Prior stroke/TIA	26 (36.1)
Atrial fibrillation	
Persistent/Permanent	38 (52.8)
Paroxysmal	34 (47.2)
CHA_2_DS_2_VASc score	4.3±1.6
HASBLED score	3.7±0.9
LVEF (%)	59.5 [50-60]
Reduced LVEF (<40%)	11 (15.3)
Intraprocedural contrast administration (ml)	82.0 [59-113]

Values are mean ± SD, n (%), or median [interquartile range]. *BMI* body mass index, *CABG* coronary artery by-pass graft, *LVEF* left ventricular ejection fraction, *TIA* transient ischemic attack

**Fig. 2:**
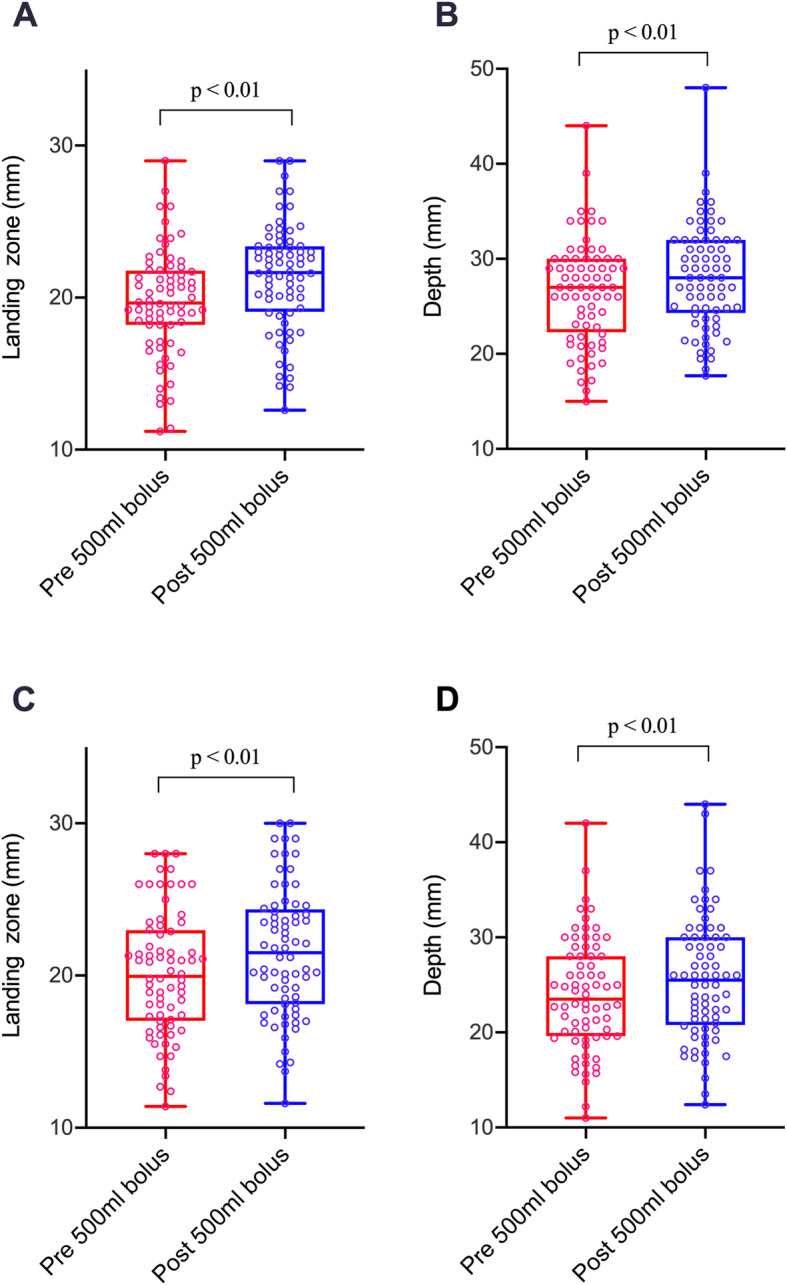
Graphical representation showing the changes in the left atrial appendage landing zone and depth, before and after volume loading, at 90 (**a/b**) and 135 degrees (**c/d**)

The maximum diameter of the LZ and depth, before and after volume loading, are detailed in Table 2, and a visual representation of the changes in the LAA LZ and depth are depicted in Fig. 2. The baseline mean LZ was 19.6 ± 3.6 mm at 90^o^, and 20.2 ± 4.1 mm at 135^o^. After volume loading, the maximum diameter measured at the LZ increased to 21.1 ± 3.6 mm at 90^o^ (*p*<0.001), and to 21.5 ± 4.3 mm at 135^o^ (*p*<0.001), which corresponded to an overall average increase of 7.0%. The baseline mean depth of the LAA was 26.5 ± 5.5 mm at 90^o^, and 23.9 ± 5.8 mm at 135^o^. Following volume loading, the mean depth increased to 28.0 ± 5.5 mm (*p*<0.001) and 25.5 ± 6.3 (*p*<0.001) at 90^o^ and 135^o^, respectively. Age, patient sex and body surface area did not have a significant impact on post bolus LAA dimensions.

**Table 2:** Maximum diameter of the landing zone and depth of the left atrial appendage, before and after administration of 500 mL IV bolus of normal saline

	Pre 500 ml bolus	Post 500 ml bolus	Delta (volume bolus - baseline)	*p* value
90^o^, landing zone (mm)	19.6 ± 3.6	21.1 ± 3.6	1.5 ± 1.0	<0.001
90^o^, depth (mm)	26.5 ± 5.5	28.0 ± 5.5	1.5 ± 1.8	<0.001
135^o^, landing zone (mm)	20.2 ± 4.1	21.5 ± 4.3	1.3 ± 1.0	<0.001
135^o^, depth (mm)	23.9 ± 5.8	25.5 ± 6.3	1.6 ± 2.0	<0.001

**Fig. 3:**
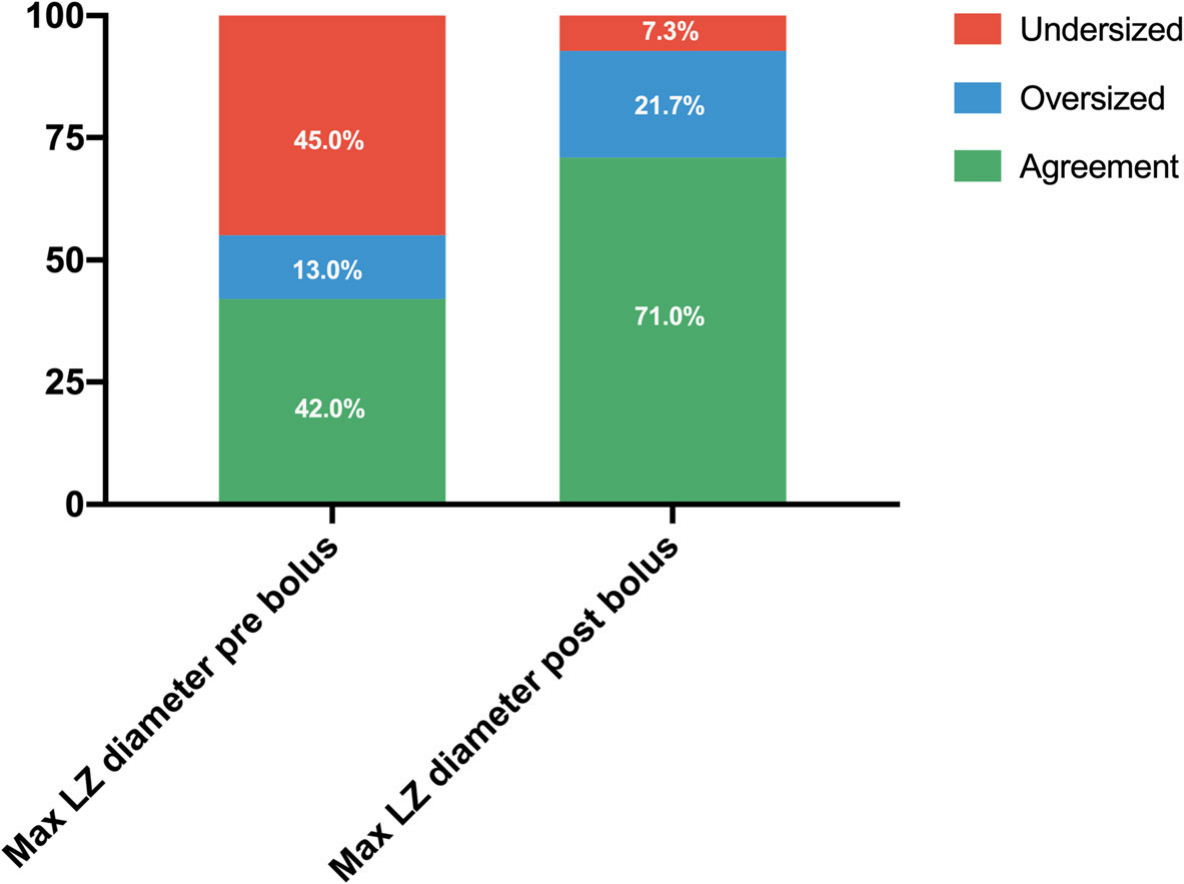
Agreement between the device size that was actually implanted and the predicted size based on the largest landing zone diameter measured pre-procedurally, before and after fluid bolus. LZ: landing zone

Successful LAAC was achieved in all but 3 patients (3/72): two due to insufficient depth and the other due to an orifice that was too small for closure. There were no cases of device embolization. All procedures were performed under general anesthesia using the Watchman (88.4%) or the ACP/Utraseal devices (11.6%). The percentage of agreement between the device size that was actually implanted and the predicted size based on the largest LZ measured pre-procedurally are shown in Fig. 3. Sizing based on pre-bolus measurements predicted the final device chosen by the operator only in 42.0% of cases, and would have resulted in device oversizing in 13.0% and undersizing in 45%. Sizing based on post-bolus measurements of the LZ significantly improved the agreement with the operator’s device choice (71.0%), and would have potentially resulted in oversizing in 21.7% and undersizing in 7.3%.

## Discussion

The results of the present study showed that the administration of an IV bolus of 500 mL of normal saline in the ambulatory setting is safe and results in significantly larger LAA dimensions. Sizing based on post volume loading measurements taken pre-procedurally significantly improved the agreement between the predicted device size determined by the manufacturers’ recommendations and the final size of the device implanted.

LAA closure has emerged as a mechanical alternative to pharmacological stroke prevention in patients who cannot tolerate oral anticoagulation [4, 5, 12]. However, in order to optimize procedural success and reduce periprocedural complications that may offset the efficacy of the procedure, appropriate sizing of the occluder device is of paramount importance. Undersizing may result in device migration or inadequate sealing of the LAA cavity, which in turn could lead to peridevice leaks, thrombus formation and embolic stroke. On the other hand, excessive oversizing may lead to physical expulsion of the device from the LAA and may cause pericardial effusion/ tamponade due to perforation [13, 14]. Baseline preprocedural TEE to screen candidates for LAA closure is performed under fasting conditions, which may reduce intravascular volume and lead to inadvertent undersizing of the LAA. We observed an average increase of approximately 1.5 mm in the LZ and depth after volume loading, confirming the notion that the LAA is a compliant structure dependent on volume status. Previous studies have shown a similar, albeit slightly higher, increase of ∼2 mm in the width and depth of the LAA after volume loading [7, 8], which is clinically relevant since it likely results in upsizing the occluder device by an entire size. The likely reason for the more modest increase in size found in the herein cohort is the fact that the aforementioned studies administered on average a higher fluid bolus (∼800–1000 ml), and relied on invasive left atrial pressure monitoring to ensure adequate volume status. In the ambulatory setting, since invasive hemodynamic monitoring is not feasible and IV fluid administration may trigger some complications such as pulmonary edema, particularly in patients with reduced LVEF, we opted to give a smaller volume of normal saline. This strategy proved to be well tolerated, and all examinations were performed without any adverse events. Moreover, the predicted device size based on the LZ dimensions taken post volume loading correlated well with the device size that was actually implanted in 71% of patients. Conversely, similarly to what has been previously reported [8], sizing based on the 2D maximum diameter of the LZ before volume loading correlated poorly with the device size finally selected (42%). It is noteworthy that the timing and optimal volume of the saline infusion should be the object of further studies. An individualized dose of saline taking into account LVEF and adapted to body surface area might be a better choice. Also, volume loading might be administered before TEE probe insertion, however, it is important to take into account that the physiological effects of a fluid bolus typically dissipates within the hour [15].

During the evaluation phase of patient suitability for LAA closure, being able to accurately define the anatomy and predict the device to be implanted is crucial as it allows for the Heart Team to make a more informed preprocedural evaluation. For instance, one of the most commonly used devices, the Watchman device, requires an implant depth equal to the orifice diameter, and thus cannot be implanted in a shallow LAA. As such, one benefit of administering a volume loading during preprocedural evaluation is to avoid contraindicating the procedure on the basis of on an incorrect depth. This is also applicable to other devices, where an incorrect measurement can erroneously contraindicate a procedure. It is of note that the use of 2D imaging to define the varied and complex morphology of the LAA may fail to depict its true dimensions, and other imaging techniques/modalities such as real time 3D-TEE and multidetector computed tomography-based LAA sizing may be more accurate methods, especially if undertaken in an euvolemic state [8, 16, 17]. Also, some authors have suggested performing the LAAC procedure with no procedural TEE guidance in order to avoid general anesthesia and facilitate patients’ recovery [9–11]. In these cases, procedural LAAC guidance is based on fluoroscopy [9] or intracardiac echocardiography [10, 11], and device sizing is mainly based on pre-procedural TEE measurements. In such cases, the strategy of volume loading during pre-procedural TEE would be key in order to provide reliable measurements and avoid complications related to inaccurate device sizing.

### Limitations

Our study has several limitations. This report consisted of an observational single-center study with a retrospective analysis and a relatively small number of patients, which limits the strengths of our results. Imaging the LAA may be challenging in some cases and it was not always possible to obtain identical images before and after volume loading. Also, observer variability was not investigated, and the lack of blinding regarding patients’ fluid status may have introduced some degree of measurement bias. However, in order to reduce interobserver variability and maintain consistency, all studies were analyzed by a single measurer and the magnitude of increase in LAA size found in our cohort was consistent with previous studies [7, 8]. Additionally, due to logistical and technical reasons, we were unable to analyze 3D data, which is in theory a more accurate method to size the LAA and might have yielded larger dimensions. Some patients might have started with an adequate volume status, which might be one of the reasons for the smaller increase in LAA dimensions found in some cases. Lastly, regarding the safety of administering a 500 ml IV fluid bolus, even though the procedure was well tolerated by all patients included in the cohort, we acknowledge that the size and design of the study does not lend itself to generalize our results since patients with heart failure and reduced LVEF were in the minority.

## Conclusions

Similar to what has been previously reported for intraprocedural LAA closure, volume loading during baseline preprocedural TEE is safe and significantly increases the LZ and depth of the LAA. This strategy improves sizing and more accurately predicts the device size that will be implanted in a subsequent intervention, which may be particularly relevant in LAAC procedures performed without TEE guidance.

## Data Availability

The datasets used and/or analysed during the current study are available from the corresponding author on reasonable request.

## Abbreviations

AF: Atrial fibrillation
IQR: Iinterquartile range
LAAC: Left atrial appendage closure
LZ: Landing zone
TEE: Transesophageal echocardiography
IV: Intravenous
